# Supplementary material for: Unveiling hidden structural patterns in the SARS-CoV-2 genome: Computational insights and comparative analysis
Source: PLoS One. 2024 Apr 4;19(4):e0298164. doi: 10.1371/journal.pone.0298164 (PMC10994416; doi:10.1371/journal.pone.0298164)
Supplement: S1 File — (ZIP) [file pone.0298164.s001.zip › S1_File.pdf]

Table 1: Predicted structures, and the SARS-CoV-2 location of each blockset. 'R' is RNAz, 'L' is Locarna, 'C' is CaCoFold.

Start	End	Blockset	Utility	Structure
1	140	RNAz_0	R	.....................................(((.....)))..........(((((.....))))).((((.......))))........((((((((.((.((((.(((.....))).)))))).))))))))............
			L	............................(((.....)))..........(((((.....))))).((((.......))))........((((((((.((.((((.(((.....))).)))))).))))))))............
			C	......(((((((.....((((((....)))))).......)))))))..........((((.......)))).((((.......))))........((((((((.((.((((.(((.....))).)))))).))))))))............
81	238	RNAz_1	R	...((((((((.((.((((.(((.....))).)))))).))))))))......................(((.(((((.((((.(((((...)))))...)))).....((((((.(((((......)))))..)))))).....)))))))).......
			L	...((((((((.((.((((.(((.....))).)))))).))))))))......................(((.(((((.((((.(((((...)))))...)))).((..((((((.(((((......)))))..))))))...)))))))))).......
			C	.....(((((((((.....)))))))))((((((.........((((.................((((..(((((.......)))))))))............)))).((((((.(((((......)))))..))))))..............))))))
179	338	RNAz_2	R	.........((((((.(((((......)))))..))))))..(((((..((((((.((......))))))))(((....)))..))))).(((..(((..(.((.((((.....))))...)).)..)))))).((..(((((.....)))))..)).
			L	......((.((((((.(((((......)))))..))))))...((((..((((((.((......))))))))(((....)))..))))((((.((........))))))((((((...((....))....))).))).((((((...))))))..)).
			C	.........((((((.(((((......)))))..)))))).........((((((.((......))))))))(((....))).((.((((....((((((.........))))))..........)))).))......(((((.....)))))....
879	1038	RNAz_9	R	((((....))))(((........))).....(((((......)))))..............((((((((...((((..(((((.((.((((((......)))))))).)))))...(((((....)))))........))))))))))))........
			L	(((...((((..(........)..))))...(((((......)))))..........))).((((((((...((((..(((((.((.((((((......)))))))).)))))...(((((....)))))........))))))))))))........
			C	.........(((((((((.........)))))))))........................((((((((...((((((((((((.((((((......))))))))))))))...(((((....)))))......))))))))))))))........
1479	1638	RNAz_15	R	.((((((((.(((((((....((((((((((.(((((....(((.(((.....)))..))).)).))))))))))))))).......)))))))......((.((((((((......(((.....))))))))))).))...))))))))......
			L	........(.(((((((....((((((((((.((((...((.(((.((((...))))..))).)).))))))))))))))).......))))))).)......(((((....(((.(((((((......))...)))))..)))...))))).........
			C	..........(((((((...((((((((((((.((((......(((.(((.....)))..)))....)))))))))))))))).......)))))))..........((((((((((...............)))))))))).....................
1579	1738	RNAz_16	R	..((.((((((((((......(((.....))))))))))))).)).........((((...((((((((.......))))))))..))))..........((((((((.((((......))))))))))))......((((((((....)))))))).......
			L	...(((((....(((.(((((((......))...)))))..)))...)))))...((((...((((((((.......))))))))..))))..........((((((((.((((......)))))))))).)).(.(((((((((....))))))))).)....
			C	...(((((((((...................((((((..................((((.((((((((.......))))))))..))))).............)))))))((((......))))..))))))))).(((((((((....)))))))))......
1679	1838	RNAz_17	R	(((((.((((......))))))))).............((((..(((((((..(((((......)))))..)).........((((((........)))))((((...((((((....))))))...))))..........)))))..))))...........
			L	.....(((((...(((.((((((((((..((((((((((((....)))))))))))..........((((........)))).(((((((........)))))((((...((((((....))))))...))))...))..))))))))))..)))...)))))
			C	.....(((((...(((((((((((((((.((((((((((((....))))))))))..........(((((((((.........)))))))))..........(((...((((((((....))))))))...)))...)).)))))))))))).))))))...)))))
2276	2435	RNAz_23	R	........((((((.......................((((((..((((((((.((...(.((((((((......))))))))...)))))))))...)).)))))))..))))))..((((.........))))))))))...(((....((((.....))))......))).
			L	.((((((((.((.......)).)).)))))).........((((((..((((((((.((...(.(((((((((....)))))))))...))))))))))...)).)))))))..)))))).(((((.........)))).).......(((.(((.(((.....)))..))).))).
			C	............((((((((((((((((........(((((((((((((((((((.........................))))))))).)))))))))))..........................)))))))))..))))))))))....................

Start	End	Blockset	Utility	Structure
2760	2919	RNAz_28	R	((((...(((((.((((((((.......))))))))))))..........(((((..((((((......))))))(((((..((((((.((((((((..((....))...)))))))).))))))....)))))...)))))....))))).......
			L	.....((((.((.((((((((.......))))))))))))))......((.(((.((((((..(((.(((.....((((((((.(((((.((((((((.(((....)))..)))))))).)))))).)..))))).))..))))))..))))))))))
			C	.....((((....((((((((.......))))))))..)))).......(((((.((((((.(((.((((......((((((..((((...(((((((((((.......)))))))))))...))))....))))))....)))).))))))))))))))
2960	3116	RNAz_30	R	..((((.......))))....(((((((....(((((.((((............(((((((.......(.((((((((..((((.....))))...)))))))).).......)))))))............))))...)))))..)))))))....
			L	..((((.......)))).((((((((((....(((((.(((((..((((((((..(((((((....((((.((((((((..((((.....))))...))))))))))))).....)))))))......)))))))).))))))))))..))))))..))))
			C	((((((((((((....))))))))))))........((((((..........((((((((((((..((((((((((((((((..((((.....))))..)))))))))))))))...))))))))))).............))))))..............
3157	3316	RNAz_32	R	..(((((..((((((((........))))))))...)))))...((((((.((.((((.(((.....(((((((.((.....)))))))))...))).))))..)).)).((((((........(((((((.....)))))))..))).))))))))....
			L	..(((((.(((((((((........)))))))))..)))))..((((.((.((.((((.(((.....(((((((.((.....)))))))))...))).))))..)).))))))(((.(((..(.((((((((...)))))))).)..))).)))......
			C	..(((((...((((((((........))))))))....)))))((((((((....((((((((........((((((((......))))))))..................)))))))).)))))))).....((((((...(((.....)))...))))))...
3211	3370	RNAz_33	R	((((.(((.....(((((((.((.....)))))))))...))).))))(((.((((((((((..((((((.((((((((...(((.....)))...)))))).)).))))))(((........))).)))))))...)))).))).............
			L	((((.(((.....(((((((.((.....)))))))))...))).))))...((((((((((((((((((((.((((((((...(((.....)))...)))))).)).))))))))........)).)))).))))))..(((((((......))).))))
			C	((((((((.....(((((((((......)))))))))......................((((((((.((((((((...(((.....)))...)))))).)).))))))))..))))))))........((((((((.......)))))))))....
4237	4396	RNAz_44	R	(((....((((....)))).(((..(((((((.(((......))).)))))))(((((.....)))))...))))))............((((((((..((((((((((((((......))))))...))))))))...........))))))))..
			L	...(((((((((...((((......)))).....))))))))..((((((((((((...(((.(........).))).....)))))))).....)))))..(((....((((....))))....)))((((((.((((....)))).))))))........
			C	...(((((((.....(((......))).......)))))))..........................((((((((....))))))))........(((((..............)))))...((((((((((((....))))))))))))).......
4737	4896	RNAz_49	R	.((((....(((((((((((((((((.((((((...))))))..................(((((((.....))))))))))))))))))......))))))))...(((((.((((....)))).))))))))))......(((((((....)))))))..
			L	(((((.((((((....(((((((((((.......(((........))).......))))))....((((((.(((.......))))))))).......))))).)))).........))))))).......((((((...))))))).(((....)))......
			C	((((.................)))).((((((((...........(((.....((((((((............((((.......)))).........))))))).....)))...................)))))))).........................
4837	4993	RNAz_50	R	.......(((((.((((....)))).))))).........(((((((....)))))))..........(((((((....).)))))).(((((...)))))...((((.((((((.((((((.....)))))).))))))).)))))................
			L	.((((....(((((((((((((((((.((((((...))))))..................(((((((.....))))))))))))))))))......))))))))...(((((.((((....)))).))))))))))......(((((((....)))))))..
			C	.........((((((((((((((((........)))))).)))).((((((((((((((((......))))))))...)))))))............)))))))((((((((((...))))))))))............(((((((....)))))))..
4934	5093	RNAz_51	R	.((((((.((((((.((((((.....)))))).)))))).)))........((((((((((.......(((..(((((((((.......))))))))).)))....(((.....))).))))).)))))........)))...................
			L	.......(((((.((((....)))).)))))......((((((((((....))))))..........((((((.......)))))).(((((...)))))...((((.((((((.((((((.....)))))).))))))).))))............)))).
			C	....(((((((((((...)))))))))))............(((((((....)))))))..........(((((.......)))))(((((((...))))))).((((.((((((.((((((.....)))))).))))))).))))................
5134	5293	RNAz_53	R	...........(((((..(((((...((((((((.....((((((((((....)))))).))))..(((..............)))))))))))............((((.(((((......(((((......)))))))))).)))))))))..)))))
			L	.((((((((......(((.(((.(((..((.((((((((.((.((((((....))))))...)))))))).)))).))..))).)))...)))........((((......))))((((((((....))))))))((((...))))............)))))))..
			C	....((((.........................(((((.........................)))))......))))....(((((((((.........(((((....)))))......)))))))))..............((((((((....))))))))

Start	End	Blockset	Utility	Structure
5234	5393	RNAz_54	R	......((((.(((((......(((((......)))))))))).))))..((((((((.......(((((.....((((((((......))))))))....))))))..))))).((((((........))))))..)).))...........
			L	............(((((...((((((.(((((.....((((((((((....)))))).))))..(((...............)))))))))))))))..))))).((((.(((((......(((((......))))))))).))))...........
			C	...........(((((((((((.(((((((((....((((((((((....))))))))))))((((((.......)))))......))))))))))..........(((((((((......(((((......)))))))))))))))))))))))))))
5434	5593	RNAz_56	R	.........((.((((....))))))....((((.((((((.((..(((.((((((((.........))))))))))..))............((((((......))))))........))))(((((((((....))))))))...))).))))..
			L	((((..(((((.((((...(((((((........))))))).((((((((((.(((((((((....))))))))).)))).))))))))....(.((((.(.((((((...(((((((...))).))))..)))))).).)))).))))))))).)))).
			C	..................(((((((........))))))).(((((((.....((((((((....))))))))).......)))))))............(((((((((.((((....))))))))..))))))..((((....))))..........
5534	5693	RNAz_57	R	.......(((.(((..(((((......((((((((....))))))))..)))))).......((((((.(((....)))..)))).)))........))))))(((((.(((((((((....)))))))).)))))....................
			L	.........((.((((....))))))....((((..((((((.((((((.((((((((.........))))))))))))).......)).)))))))(((((......)))))...(((((......((((((((....))))))))..)))))))))..
			C	((((((((((...........((((((..........(((((((((((.((((((((.........)))))))))))))).......))))))))).............))))))...................))))))))))...............
5634	5793	RNAz_58	R	...((((((.(((((((((....))))))))).))))))....................((((((((...((((.((.(((((......))))))))))))))))))).............((((((((....))))))))..............
			L	(((........)))...(((((......((((((((....))))))))..)))))).(((((.((((((.(((....)))..)))).)))..........))))(((((.(((((((((....)))))))).)))))....................
			C	.........................((((((((....))))))))..............((((((((((.......))))))).))).....((((((((.((((((.(((((((((....)))))))))).)))))))..))))))))).........
6134	6293	RNAz_63	R	.(((((...............((((.......((.(((((((.(((((....))))).))))))).)).(((.....))))....)))))..((((((((....)))))))).(((....))).)))))((((((.((.(......))).)))))))....
			L	((.(((.....((((..((((((.((((.(((((((..(((.....)))..)))))))))))....)))))))..))).)......))).))....(((...(((((((.......((...((.........))...)).....)))))))...)))...
			C	............((...((((...((((.(((((((.(((.......))).))))))))))).......))))...))..................(((((.(((((((...........(....)...................))))))).)))))...
6534	6693	RNAz_67	R	.......((((((((....((((.((((((((....))))))))))))).......))))))))...............(((((((((.(((......)))..(((((((......))))))).)))))))))..........................
			L	..((((.....))))((((((.(((....((((....))))..((((....))))(((((((((..(((.......)))))))))))..(((((..((.....)).))))))))).)))))))(((((((((....)))))))))..............
			C	...............((((((..........................)))))))..((((((((((............))))))))))................(((((((((((((((....((((((....))))))))))))))).))))))).
13314	13470	RNAz_134	R	..............................(((....)))...................((((((((((((((((........)).))))))))))....((((((.....))))))............)))).........................
			L	..........((((..(((((((...(((((...)))))...........)))))))..)))).(((((((....((.(((...(((.((((......(((((.........)).)))......)))))))))))..)))))))...................
			C	..........................(((((....)))))..(..)..................
18619	18769	RNAz_188	R	(((((.(((......((((((((....))))))))..((((((..(((.........))).)))))).........)))...)))))...............(((((((.....(((...((((((.....))))))..))).)))))))....
			L	...................((.((((((.((((.(((.(((((((.(((((.....)))))..))).........)))).)))...)))))..))))))))..(((((((((...))).))))))..(((.((((.........)))).))).
			C	...............((((((.((..)).)))))).....................(((((((.......)))))))..............(((((.......)))))...
19296	19448	RNAz_195	R	......................................(((...((.(((((.((((.(((...(((.....)))...))))))).))))).)).)))..........((((......((((.....))))......)))).........
			L	(((((((....)))))))............(((((((.....)))))))(((((....)))))........(((...(((((...((((((.((((((.......))))))............))))))).)))))..)))............
			C	(((((((....)))))))..((((((((((.((((((((((((.......))))))...)))))))).)))))))))))......................

Start	End	Blockset	Utility	Structure
20986	21142	RNAz_211	R	((((((.(((((((.....))))))).)))))).(((............((((..(((((((.(((((......)))))........)))))))..))))((((((..........................))))))..)))...........
			L	(((((((((((((..(((......)))..)))..(((((((......)))))))(((((((((((((.........)))......)))))))))))....((((((.(((((((.....))))))).))))))...........)))))))))))...
			C	.....................................((((((((((..................)))))))))).......(((...........((((((((((((((.......))))))))))))))...................))))..
21892	22051	RNAz_220	R	..(((((.............................(((.((((..(((((...((((((......))))))...)))))...)))).))).((((((.............))))))...)))))..........(((((((.(.....).))))))).
			L	.........(((...)))...((((.....((((((((.......))))))))...))))((.(((((((..(((((.((....(((((((((((((((.....)))).))))))..........)))))).))))))))))).)).............
			C	((((((((((((((((..............(((((((.........)))))))........))))))))))))))))...........((((((((((........))))))))))((((....))))..................................
21992	22149	RNAz_221	R	.....((((((........))))))...............(((.....(((((((.(((((((((........)))....))))))))))))))((((((((......))).))))........)))..((((((........))))))............
			L	.((((((.......((((....))))........(((((..((..(((((....(((((((....)))))))..))))).))...))).)).(((((((..........)))))))...))))))........(((((((.(.....).))))))).
			C	..(((((................((((((((((((...(((((((((((((((((.(((((((....))))))).))))))))).)))))))).))))))))))))).............)))))((((((((((...........)))))))))).
25394	2540	RNAz_250	R	(((((...((((((...((((((.(((((.....(((.....))).))))))..))))))))))))........(((.....))))))))............(((((.....((((...((.....)).))))...))))).................
			L	......(((((((..(((((((((((.........)))))..))).)))..)))))))..............(((((......)))))..........((((((....))).)))..((((((.(((((((.(.........))).))))))..))))))..
			C	.........(((((((((((((((((.......))))))).))))))))))......((((((.(((((.......))))).)))))).................((((((....))))))....................(((........))).
25579	25737	RNAz_252	R	(((((((.........(((((((..................)))))))).....((((((((((((......)))))))))))...))))))...(((((.....((((((......)))))......((((......))).)))))...........
			L	...(((((.....(((((.(((.....)).)))))).))))).............(((((..((....(.(((..(((......)))..))).)....))..)))))....(((.(((((((..................)))))))...))).....
			C	................((((((((..((((.......))))......................))))))))).(((((.....................)))))....((((((((((.((........)).))))))))))..............
25678	25835	RNAz_253	R	......(((((......))))).................((((((.(..........).)))))).......(((((.((((((((((.....)))))))))))))).........(((((((((.....)))))))))...................
			L	...((((..((.....((((((((..................)))))))......((((((((((((....)))))))))))).......(((....(((.(((..((((((......))))).))).)))...)))......))))))...........
			C	............((((((((((.((........)).)))))))))).........((((((((((((....)))))))))))...(((((.((((((((.(((((.......))))).)).))))))))))).........................
25777	25935	RNAz_254	R	.(((..(((((......(((((((((.....)))))))))).......((((((((.(.(..((((((((((.....))))))))))..).).))))))))....)))))....((((((((......))))))))....))))....
			L	.((((.((((((......))))))......(((......)))((((((.(...........).)))))).......(((((.((((((((((.....))))))))))))))........((((((((((...)))))))))))...))))..............
			C	.....((((((......))))))................(((((................)))))).......((((((.(((((((.......)))))))))))))).........(((((((((.....)))))))))...................
25876	26019	RNAz_255	R	.................(((((....((((((((......)))))))))))))).((((((.....(((((((((((((((.....))))))))))))...)))))).................(((((.((((.....)))).)))))..))))))..
			L	.................((((((((((...))))))))))).......((((((((......((((((((((...))))))))))......))))))))...(((((((....(((((((......))))))))))))))).....
			C	................(((((((((.....)))))))))........(((((((((......(((((((.....)))))))).....))))))))))......((((.....((((((((......))))))))....))))...
25960	26118	RNAz_256	R	..((((((((.....................(((((....)))))..(((((((((..((((((....((((.........))))..))))))..))))))))).))))).))).....
			L	.................(((((((....(((((((......)))))))))))))))((((((.....((((((((((((((((.....))))))))))))...))))))..(((.........)))((((((.(((((...))))).)))))).))))))..
			C	..(((((((((..((((((((((.((((((((((((......)))))))))).))).)))))))))))..((((((((((((((((.......))))))))))...))))))............)))))))))((((((((((....)))))))))).........

Start	End	Blockset	Utility	Structure
26059	26207	RNAz_257	R	................................(((.((((((((..(((((....((((....))))..))))))..))))))))).)))...........((((........))))........(((((((......)))))))
			L	...(((((((((.(((((.((......((((((.(((((....))))).))))))).......))))))).)))..........(((.((((((((((.((((((....((((....))))..)))))).)))))))))).)))....))))))
			C	.((((((((((((((((..((((...(((..(((((....)))))..)))...))))..)))))))))))))))))..........(((((((((...............(((((......)))))).............)))))))))...........
26397	26546	RNAz_261	R	....((((.(((((.....(((...((((((((.........................))))))))............................(((((((....))))))) ((((((((.....)))))))))))....))))))))).
			L	...........................((((......(((.((((((((......)))))))..)))..........(((((((((((.(((.((.........)).)))))).)))))).)))..............))))..........((((....))))
			C	.((((((((((((((((((((..............................(((((...(((((............)))))...)))))...((((((((((...................))))))))))..))))))))))))))))))))).
26443	26590	RNAz_262	R	....(((((((.(((((......))))).))))))))..........................((((((.....))))))...
			L	....((((.(((((.........(((((((((((((((........))))))....)))))))).)))....................((((((((....)))))))) (((((((.....))))))).........))))))))).
			C	............((((((((((...(((((((..............................)))))))...............................((((....))))))))))))))................................
27276	27428	RNAz_270	R	..........................((((.....(((((((((((((...((((((..(((((.....)))))..))))))...)))))))....(((((((((((....)))))))))))))))..(((((((.((..........))))))))))))...
			L	....((..(((((((((((.............)))))))............(((((((.(((((((....))))))).)))).)))........((((.(((((.(((((...................))))).))))..))))....)))..))..
			C	......................(((((((((((.......))))))))))).......(((((((((((((....)))))))))))))..((((((......(((((.........)))))......................)))))).................
27766	27922	RNAz_275	R	..............(.(((((((((..(((((.((....)).)))))..)))))......((((((((((((((((((......)))))))).)))))))..))))).....(((((((........)))))))...))))).).....
			L	............((.........))......((((......))))...(..((((((.(((((.........(((((.((((((......)))))).)))))))))))))))))..)........((((.(..(((((.((....)).)))))..)))))......
			C	....................((((((...(((((.((((.................................(((((((.((((....)))).))))))) ((((......))))....)))).)))))....)))))).(((((((......)))))))...

Table 2: Base pair, Hamming, and Levenshtein distances between predicted structures.

	**Base pair**			**Hamming**			**Levenshtein**		
**Blockset**	DistR-L	DistR-C	DistL-C	DistR-L	DistR-C	DistL-C	DistR-L	DistR-C	DistL-C
RNAz_0	58	67	67	70	81	79	10	30	27
RNAz_1	2	62	64	4	66	66	4	60	64
RNAz_2	33	32	33	32	40	36	29	81	77
RNAz_9	15	24	25	14	26	34	15	33	33
RNAz_15	43	17	30	35	32	37	33	32	33
RNAz_16	93	47	80	45	30	51	28	41	47
RNAz_17	59	72	23	50	58	26	65	74	26
RNAz_23	88	77	85	57	68	77	34	67	85
RNAz_28	43	50	25	42	54	28	37	46	27
RNAz_30	27	65	72	34	52	58	34	55	62
RNAz_32	23	58	61	18	48	50	20	49	50
RNAz_33	38	46	46	22	56	50	28	57	42
RNAz_44	93	83	40	82	100	46	85	86	50
RNAz_49	88	76	62	74	96	70	65	81	74
RNAz_50	95	93	72	82	70	72	81	85	51
RNAz_51	89	88	27	82	74	28	72	72	23
RNAz_53	92	75	63	76	94	78	75	85	75
RNAz_54	81	89	46	64	80	40	68	86	40
RNAz_56	103	84	85	67	70	57	73	72	51
RNAz_57	92	80	48	94	94	62	84	81	63
RNAz_58	88	85	29	82	64	44	89	87	48
RNAz_63	90	80	18	76	84	36	73	70	34
RNAz_67	89	77	58	60	86	64	77	85	76
RNAz_134	65	31	46	72	54	74	68	52	71
RNAz_188	84	61	63	69	81	84	74	71	68

	Base pair			Hamming			Levenshtein		
**Blockset**	DistR-L	DistR-C	DistL-C	DistR-L	DistR-C	DistL-C	DistR-L	DistR-C	DistL-C
RNAz_195	68	59	45	69	65	54	83	76	52
RNAz_211	88	64	52	67	105	88	64	81	73
RNAz_220	82	73	55	82	78	62	88	73	75
RNAz_221	74	90	96	88	82	73	65	77	67
RNAz_250	82	77	71	74	73	65	65	58	63
RNAz_252	75	68	67	71	81	62	61	71	65
RNAz_253	74	77	81	56	59	69	55	69	43
RNAz_254	87	77	14	83	85	26	65	76	26
RNAz_255	86	81	33	76	66	26	50	42	22
RNAz_256	82	86	60	79	81	42	89	93	49
RNAz_257	83	76	93	84	93	77	85	89	55
RNAz_261	67	76	76	98	69	107	82	68	84
RNAz_262	59	40	49	92	65	69	84	54	60
RNAz_270	85	78	51	88	70	80	84	72	66
RNAz_275	78	80	78	88	78	66	57	64	71

Table 3: Dunn's post hoc test p-values for all comparisons between pairs of distances. Each row and column reflects the distance between the distances of each different dataset. Z - Ziesel (this work), A - Andrews, H - Huston, L - Li and M - Manfredonia datasets.

	**Z-A**	**Z-H**	**Z-L**	**Z-M**	**A-H**	**A-L**	**A-M**	**H-L**	**H-M**	**L-M**
**Z-A**	1	1	0.17	1	1	$1.2 * 10^{-3}$	1	1	$2.2 * 10^{-2}$	1
**Z-H**		1	$2.3 * 10^{-3}$	1	1	$3.8 * 10^{-6}$	1	$3.5 * 10^{-2}$	0.89	$3.4 * 10^{-2}$
**Z-L**			1	$2.5 * 10^{-4}$	$7.8 * 10^{-2}$	1	$2.8 * 10^{-2}$	1	$8.1 * 10^{-9}$	1
**Z-M**				1	1	$2.3 * 10^{-7}$	1	$5.4 * 10^{-3}$	1	$5.2 * 10^{-3}$
**A-H**					1	$4.1 * 10^{-4}$	1	0.66	$5.3 * 10^{-2}$	0.65
**A-L**						1	$4.8 * 10^{-6}$	1	$7.0 * 10^{-13}$	1
**A-M**							1	$4.1 * 10^{-2}$	0.80	$4.0 * 10^{-2}$
**H-L**								1	$5.8 * 10^{-7}$	1
**H-M**									1	$5.6 * 10^{-7}$
**L-M**										1
